# Engineered Bi-Specific AChR CAAR T Cells for Selective Elimination of Myasthenia Gravis B Cells

**DOI:** 10.21203/rs.3.rs-9094407/v1

**Published:** 2026-03-20

**Authors:** Niels von Wardenburg, Gregorio Spagni, S. Momsen Reincke, Stephanie Wernick, Hans-Christian Kornau, Viktoria Zinnow, Amelya Keles, Lucie Y. Li, Marie A. Homeyer, Sonja Blumenau, Maria Stecklum, Dietmar Schmitz, Andreas Pelz, Valentina Damato, Nicholas Sanderson, Tobias Derfuß, Minh C. Pham, Kevin C. O’Connor, Andreas Meisel, Harald Prüss

**Affiliations:** 1 Department of Neurology with Experimental Neurology, Charité – Universitätsmedizin Berlin, corporate member of Freie Universität Berlin and Humboldt-Universität zu Berlin, Berlin, Germany; 2 German Center for Neurodegenerative Diseases (DZNE) Berlin, Berlin, Germany; 3 Berlin Institute of Health at Charité Universitätsmedizin – Berlin, Berlin, Germany; 4 Department of Neurosciences, Psychology, Drug Research and Child Health (NEUROFARBA), University of Florence, Florence, Italy; 5 Neuroscience Research Center (NWFZ), Charité – Universitätsmedizin Berlin, corporate member of Freie Universität Berlin and Humboldt-Universität zu Berlin, Berlin, Germany; 6 Experimental Pharmacology and Oncology Berlin-Buch GmbH, Berlin, Germany; 7 Neurologic Clinic and Policlinic and Research Center for Clinical Neuroimmunology and Neuroscience, Departments of Medicine, Biomedicine, and Clinical Research, University Hospital Basel, University of Basel, Basel, Switzerland; 8 Department of Immunobiology, Yale University School of Medicine, New Haven, CT 06511, USA; 9 Department of Neurology, Yale University School of Medicine, New Haven, CT 06511, USA; 11 Neuroscience Clinical Research Center, Berlin, Germany; 12 Center for Stroke Research, Berlin, Germany; 13 These authors contributed equally; 14 Lead Contact

**Keywords:** CAAR T cell, autoimmunity, cell therapy, myasthenia gravis, acetylcholine receptor antibodies, chimeric autoantibody receptor, T cell

## Abstract

Myasthenia gravis (MG) is an autoimmune neuromuscular disorder caused by autoantibodies targeting the nicotinic acetylcholine receptor (nAChR), which can lead to severe disability and life-threatening crises. Current therapies rely on broad immunosuppression and fail to achieve sustained remission in the majority of patients. Here, we report the development of AChR chimeric autoantibody receptor (CAAR) T cells engineered to selectively eliminate autoreactive B cells producing anti-AChR autoantibodies. T cells were co-transduced with CAARs expressing extracellular domains of the AChR α1 or β1 subunits, enabling recognition of a broad range of pathogenic antibodies. AChR CAAR T cells selectively secreted effector cytokines upon activation, and efficiently lysed target cells. *In vivo*, they depleted pathogenic B cell lines and reduced autoantibody levels in the circulation and at the neuromuscular junction. These findings establish AChR CAAR T cells as a precision immunotherapy with the potential to achieve durable remission in refractory MG.

## INTRODUCTION

Acetylcholine receptor autoantibody-positive myasthenia gravis (AChR MG) is a chronic autoimmune neuromuscular disease caused by pathogenic autoantibodies targeting the nicotinic acetylcholine receptor (nAChR). These autoantibodies impair neuromuscular transmission, leading to fluctuating muscle weakness and, in severe cases, life-threatening myasthenic crises [1]. The nAChR is a heteropentameric ion channel essential for synaptic signaling at the neuromuscular junction. Decades of research have established the α1 subunit as the dominant autoantigen in AChR MG [2–4], while recent findings implicate AChRβ1-directed autoantibodies as additional contributors to disease pathogenesis [5, 6].

Pathogenic AChR autoantibodies act through receptor internalization, interference with channel activity, and complement-mediated lysis [7, 8]. Current treatments ranging from B cell depletion and antibody removal to complement inhibition can alleviate symptoms but remain largely non-selective. A significant subset of patients is treatment-refractory [9], and many fail to achieve durable disease control [10, 11]. Moreover, chronic immunosuppression can cause treatment-limiting side effects and increases susceptibility to severe infections, underscoring the need for more precise and antigen-specific therapies.

The emergence of chimeric antigen receptor (CAR) T cell therapy has transformed cancer immunotherapy and is now being explored for autoimmune diseases [12]. Case reports and early clinical studies on the use of CD19- or/and BCMA-targeted CAR T cells in refractory AChR MG have shown encouraging efficacy [13–19]. However, these approaches cause broad B cell or plasma cell depletion, removing the established, protective, B cell compartment, which often results in hypogammaglobulinemia and relevant infection risk [20].

Building on previous preclinical studies demonstrating the successful development of chimeric autoantibody receptor (CAAR) T cells for autoimmune diseases, we here present AChR CAAR T cells engineered to selectively eliminate autoreactive B cells in MG [21–23]. Our AChR CAAR T cells incorporate the extracellular domains of either the AChRα1 or AChRβ1 subunit of nAChR fused to 4–1BB and CD3ζ signaling domains, enabling specific recognition, and killing of AChR-reactive B cells. We demonstrate that AChR CAAR T cells selectively target pathogenic B cells *in vitro* and *in vivo*, providing proof-of-concept for a targeted and potentially curative therapy for AChR MG.

## RESULTS

### Myasthenia gravis patient-derived autoantibodies bind to AChR CAAR constructs expressed on primary human T cells

**Fig. 1.**
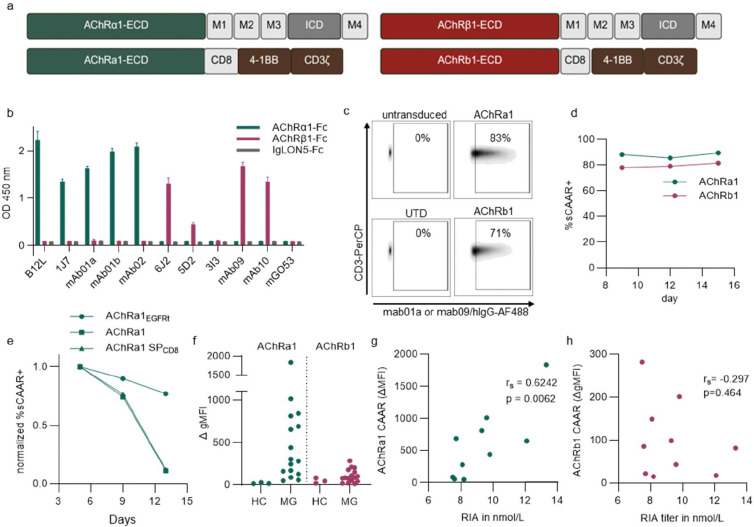
Patient-derived anti-AChR autoantibody bind to CAAR constructs (a) Schematic diagram of AChR⍺1 and AChRβ1 subunits (top) and respective CAAR constructs (bottom). Extracellular domains (ECD) were fused to the CD8 transmembrane domain followed by intracellular 4–1BB costimulatory domain (brown) and CD3ζ activating domain (brown). M1, M2, M3 and M4 represent transmembrane domains. ICD, Intracellular domains. Created with BioRender.com (b) Patient-derived recombinant subunit-specific autoantibodies were tested for binding to soluble CAAR-ECDs fused to an Fc-domain by ELISA. Antibodies were applied at 10 ng/mL. IgLON5-Fc served as negative control antigen. (c) Expression of AChRa1 CAAR and AChRb1 CAAR in primary human T cells was validated by flow cytometry. (d) Surface expression (sCAAR+) of AChRa1 and AChRb1 CAARs by primary human T cells was assessed by flow cytometry on day 9, 12 and 15 during ex vivo expansion. (e) Surface expression (sCAAR+) normalized to CAAR expression on day 5 was analyzed by flow cytometry for AChRa1 CAAR co-expressing EGFRt (AChRa1EGFRt) and not co-expressing EGFRt (AChRa1) with the endogenous signal peptide and for AChRa1 CAAR with the CD8 signal peptide as in [25]. (f) IgG binding of MG and healthy control (HC) serum to AChRa1, AChRb1 or N1ECD CAAR expressed on Jurkat cells was assessed by flow cytometry. Scatter plots indicate geometric mean fluorescence intensity (gMFI) of EGFR+ cells minus gMFI of EGFR-cells (ΔgMFI). Cut-off for binding was defined based on the mean ΔgMFI of control samples + 3 SDs. See also Supplementary Figures 1c and 1d. (g and h) Correlation of serum binding to AChRa1 CAAR (g) and AChRb1 CAAR (h) measured by flow cytometry with binding to the full AChR as measured by RIA. Statistical analysis was performed by simple linear regression; r= Spearman’s correlation coefficient. See also Supplementary Figures S1i and S1j.

The adult nAChR is a heteropentameric receptor composed of two α1, one β1, one δ, and one ε subunit. For CAAR design, we focused on the α1 and β1 subunits, which represent the principal autoantibody targets and the main disease drivers in AChR MG [2, 5, 6, 24, 25]. The extracellular domains (ECDs) of both subunits were fused via a short linker to second-generation CAR signaling domains containing the 4–1BB co-stimulatory domain, chosen to enhance T cell persistence and reduce relapse risk (Fig. 1A).

To screen for AChR autoantibody binding to AChRα1 and AChRβ1 ECD, we tested binding of MG patient-derived monoclonal autoantibodies to fusion proteins comprising the respective ECD linked to rabbit Fc using ELISA, with IgLON5-Fc as a negative control (Fig. 1b) [5, 6, 26]. As summarized in Table S1, the selected antibodies covered the known spectrum of pathogenic properties of AChR autoantibodies. All autoantibodies reactive to AChRα1 demonstrated binding to the AChRα1-Fc chimera (Fig. 1b). B12L, mab01b, and mab02 showed similar binding strength, while 1J7 and mab01a showed slightly reduced binding (Fig. 1b). Among AChRβ1-reactive autoantibodies, three of five showed strong binding to the AChRβ1-Fc chimera, while autoantibody 5D2 displayed reduced binding and 3I3 showed no binding (Fig. 1B). In binding assays using AChR-expressing HEK cells, 5D2 exhibited lower binding than 3I3 [5], suggesting that the epitope recognized by 3I3 may involve the neighboring δ subunit.

We next verified CAAR expression on primary human T cells by measuring binding of AChRα1-reactive mab01a or AChRβ1-reactive mab09 autoantibodies via flow cytometry (Fig. 1c). A truncated version of the epidermal growth factor receptor (tEGFR) was integrated after a cleavage site as a transduction marker and for enrichment of transduced T cells [27]. In contrast to a previous study on the development of AChRα1 CAAR T cells [28], we did not observe downregulation of CAAR expression up to 15 days post-transduction (Fig. 1d). We hypothesized that improved expression is mediated by usage of the endogenous signal peptide instead of the CD8 signal peptide that was used in the previous study [28]. Surprisingly, sustained CAAR expression was mediated by co-expression of EGFRt (Fig. 1e). To indirectly assess tonic signaling, we monitored cell size during ex vivo expansion (Supplementary Fig. 2a). Upon activation with CD3/CD28 beads, AChR CAAR T cells transiently increased in size but returned to baseline levels similar to CD19-CAR T cells and untransduced controls, suggesting an absence of chronic tonic signaling that could be mediated by CAAR dimerization.

To determine recognition of AChR by polyclonal autoantibodies in patient sera, we generated Jurkat cells expressing AChR CAAR constructs (Supplementary Fig. 1b, 1c) and analyzed serum binding of two MG cohorts (Berlin, Germany, and Florence, Italy) by flow cytometry. Clinical information is summarized in Supplementary Table 2. In the first assay (Fig. 1e–g), we tested 16 sera with high AChR autoantibody titers (>10-fold above positivity cutoff by ELISA or RIA). All sera bound to the AChRa1 CAAR, whereas 5 of 16 sera (31%) bound to AChRb1 CAAR (Fig. 1e). For ten samples, corresponding AChR autoantibody levels measured by RIA were available, enabling correlation analyses (Fig. 1f, g). Binding of patient sera to the native AChR correlated strongly with binding to the AChRa1 CAAR (Fig. 1f), but not to AChRb1 CAAR.

In a second assay, we selected 25 sera spanning low, medium, and high binding to full-length AChR expressed in HEK cells. As expected, CAAR binding was lower in this cohort. Nine sera (36%) bound AChRa1 CAAR (Supplementary Fig. 1e, 1g and 1h) and 3 (12%) bound AChRb1 CAAR (Supplementary Fig. 1f, 1g and 1h), with four samples (16%) recognizing both CAARs (Supplementary Fig. 1h). Binding to full-length AChR correlated moderately with AChRa1 CAAR binding (Supplementary Fig. 1i) and not significantly with AChRb1 CAAR binding (Supplementary Fig. 1j).

Together, these results demonstrate that AChRa1 CAAR T cells capture the principal autoantibody specificity in MG, while AChRb1 CAAR T cells recognize a smaller but distinct subset of patient autoantibodies.

### AChR autoantibody expressing cells induce activation and proliferation of AChR CAAR T cells

To assess the functional activity of AChR CAAR T cells, we generated K562 and Nalm6 cells expressing membrane-bound MG autoantibodies targeting either the α1 or β1 subunit, thereby mimicking human anti-AChR B cells. The density of surface immunoglobulin on these engineered cells was within the physiological range observed on primary B cells (Supplementary Fig. 2).

**Fig. 2.**
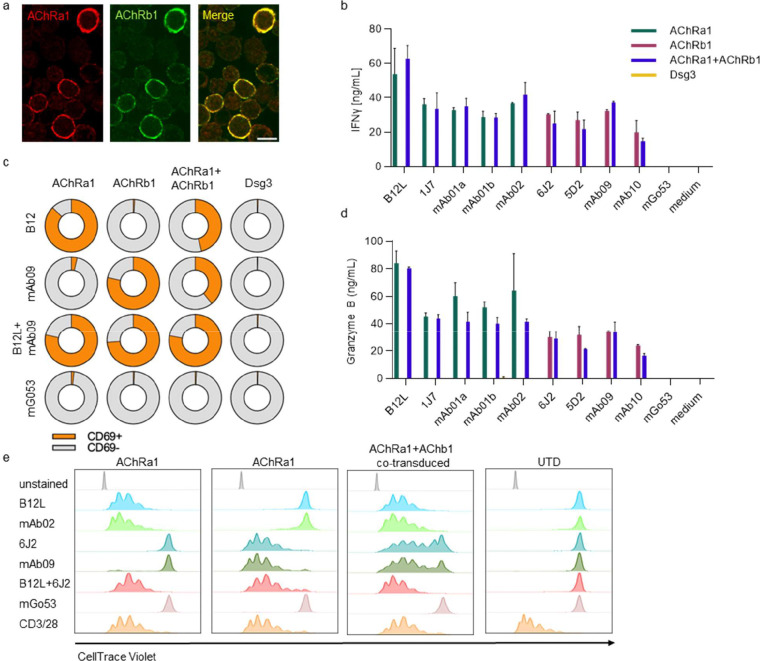
AChR autoantibody expressing cells induce CAAR T cell activation and proliferation Donor-derived CAAR T cells expressing AChRa1, AChRb1, or co-transduced AChRa1+AChRb1 were co-cultured with K562 cells engineered to express AChR⍺1- or AChRβ1-specific autoantibodies as surface B cell receptors (BCRs), to assess T cell activation and proliferation. See also Supplementary Figures 2A. (a) Confocal images of co-transduced Jurkat cells showing overlapping surface expression of AChRa1 CAAR and AChRb1 CAAR. Bar indicates 10 μm. (b, d) After 24 hours of co-culture, supernatants were collected and analyzed by ELISA for IFNγ and granzyme B (GrB) levels. Bars represent mean ± SD of triplicate cultures. Similar results were obtained using T cells from two additional healthy donors. See also Supplementary Figures 3a–d. (c) Donut charts showing the percentage of CAAR T cells expressing the early activation marker CD69 after 20 hours of co-culture, as determined by flow cytometry. Similar results were obtained using T cells from two additional healthy donors. See also Supplementary Figures 3e–g. (e) Proliferation of CAAR T cells assessed by dilution of CellTrace Violet dye after co-culture with target cells, analyzed via flow cytometry.

After validation of co-expression of AChRa1 and AChRb1 CAAR T cells in Jurkat cells by confocal microscopy (Fig. 2a), we conducted co-culture assays to evaluate the secretion of effector molecules interferon gamma (IFNγ) and granzyme B (GrB), the expression of the early T cell activation marker CD69, and the proliferation of AChR CAAR T cells. All AChRα1 and AChRβ1 target cells tested induced secretion of IFNγ and GrB by AChRa1 and AChRb1 CAAR T cells, respectively (Fig. 2b, 2d and Supplementary Fig. 3a, 3c). Co-culture of K562 cells expressing the B12L autoantibody with AChRa1 CAAR T cells resulted in the highest levels of IFNγ and GrB production (Fig. 2b, 2d and Supplementary Fig. 3a–d). T cells co-transduced with both AChRa1 and AChRb1 CAARs produced amounts of cytokines comparable to single-transduced CAAR T cells (Fig. 2b, 2d and Supplementary Fig. 3a–d), supporting the feasibility of CAAR co-transduction. K562 cells expressing an irrelevant antibody (mGo53) did not induce cytokine secretion, demonstrating the specificity of the AChR CAAR T cells (Fig. 2b, 2d and Supplementary Fig. 2a–d). Additionally, T cells expressing a desmoglein-3 (Dsg3) CAAR[21] – a clinical-stage CAAR – showed no response to AChR target cells, confirming target specificity (Fig. 2b, 2d and Supplementary Fig. 3a–d).

Consistent with cytokine release data, AChRa1 and AChRb1 CAAR T cells upregulated the early activation marker CD69 following target cell encounter (Fig. 2c, and Supplementary Fig. 3f, 3g). More than 75% of AChRa1 or AChRb1 CAAR-transduced T cells were CD69-positive after co-culture with K562 cells expressing their corresponding targets, i.e. B12L or mab09 autoantibodies respectively (Fig. 2c, and Supplementary Fig. 2f, 2g). Co-transduced T cells displayed lower CD69 induction when exposed to either antigen alone but reached equivalent activation levels when both target cells were present, likely reflecting the proportion of T cells expressing both CAARs (Fig. 2c, and Supplementary Fig. 3f, 3g). Control conditions elicited minimal activation.

As target cell-induced proliferation is a surrogate for CAAR T cell persistence in clinical application, we next evaluated the proliferative capacity of AChR CAAR T cells upon target cell encounter. Both AChRa1 and AChRb1 CAAR T cells expanded robustly upon stimulation with their respective target cell lines (B12L and mAb02, or 6J2 and mAb09), exhibiting dye dilution comparable to CD3/CD28 bead activation (Fig. 2e). Bi-specific CAAR T cells proliferated efficiently in response to either individual or combined target cell populations.

### Selective cytolysis of AChR autoantibody expressing cells

**Fig. 3.**
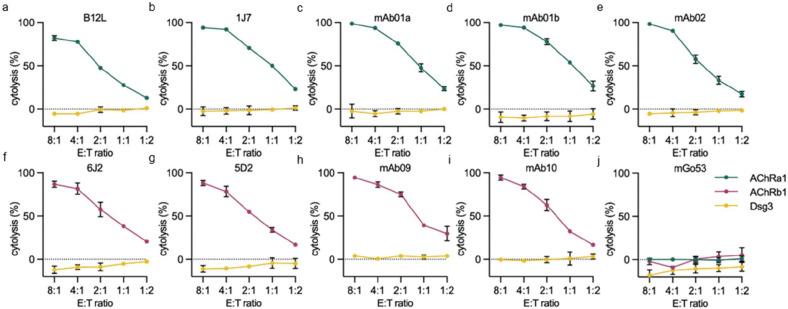
AChR CAAR T cells eliminate target cells *in vitro*. AChR CAAR T cells of Donor D8 were incubated with firefly luciferase (ffluc)-expressing target cell lines for 16–20 hours, followed by measurement of specific cytolysis via luciferase activity. Mean values ± SD of triplicate cultures are shown. Dsg3 CAAR T cells were included as negative controls. Similar results were obtained with CAAR T cells from two additional healthy donors. (a–e) AChR CAAR T cells co-cultured with K562 cells expressing AChRα1-reactive autoantibodies as BCR. E:T ratio, Effector-to-Target ratio. (f–i) AChR CAAR T cells co-cultured with K562 cells expressing AChRβ1-reactive autoantibodies as BCRs. (j) K562 cells expressing mGo53, an irrelevant antibody, served as negative controls. See also Supplementary Figure 3.

We next evaluated the cytolytic activity of AChRa1 and AChRb1 CAAR T cells using a luciferase-based killing assay. To this end, K562 target cells were engineered to express firefly luciferase, enabling quantification of cell lysis [23]. Both AChRa1 (Fig. 3a–e, and Supplementary Fig. 4a, 4b) and AChRb1 CAAR T cells (Fig. 3f–i, and Supplementary Fig. 4a, 4b) showed efficient killing across all corresponding target cells tested. Notably, substantial target cell killing was observed even at the low effector-to-target (E:T) ratio of 1:2, underscoring the high functional potency of AChR CAAR T cells (Fig. 3a–i, and Supplementary Fig. 4a, 4b). Previous studies have demonstrated highly variable binding affinity and pathogenic properties of the AChR MG autoantibodies used here (summarized in Supplementary Table 1) [5, 6]. However, AChR CAAR T cells achieved comparable cytolytic efficiency (Fig. 3a–i, and Supplementary Fig. 4a, 4b), suggesting that effective killing is largely independent of the B cell receptor (BCR) binding strength.

Specificity of the cytotoxic response was confirmed through multiple negative controls: no lysis was detected against K562 cells expressing the irrelevant antibody mGo53 (Fig. 3j, and Supplementary Fig. 4a, 4b), and Dsg3-targeting CAAR T cells failed to kill AChR autoantibody-expressing targets (Fig. 3a–i, and Supplementary Fig. 4a, 4b).

These findings demonstrate that AChR CAAR T cells mediate robust, antigen-specific cytotoxicity against AChR autoantibody-expressing cells.

### Soluble AChR autoantibodies do not impair killing efficiency but mediate low-level cytolysis of Fc receptor (FcR)-expressing cells.

To assess how circulating AChR autoantibodies might affect killing of AChRa1 and AChRb1 CAAR T cells or bi-specific co-transduced CAAR T cells, we performed in-vitro assays using mixtures of AChR monoclonal autoantibodies at concentrations within, or exceeding, the range reported in MG patients (5–25 μg/mL) [29, 30].

#### Soluble antibody blockade.

**Fig. 4.**
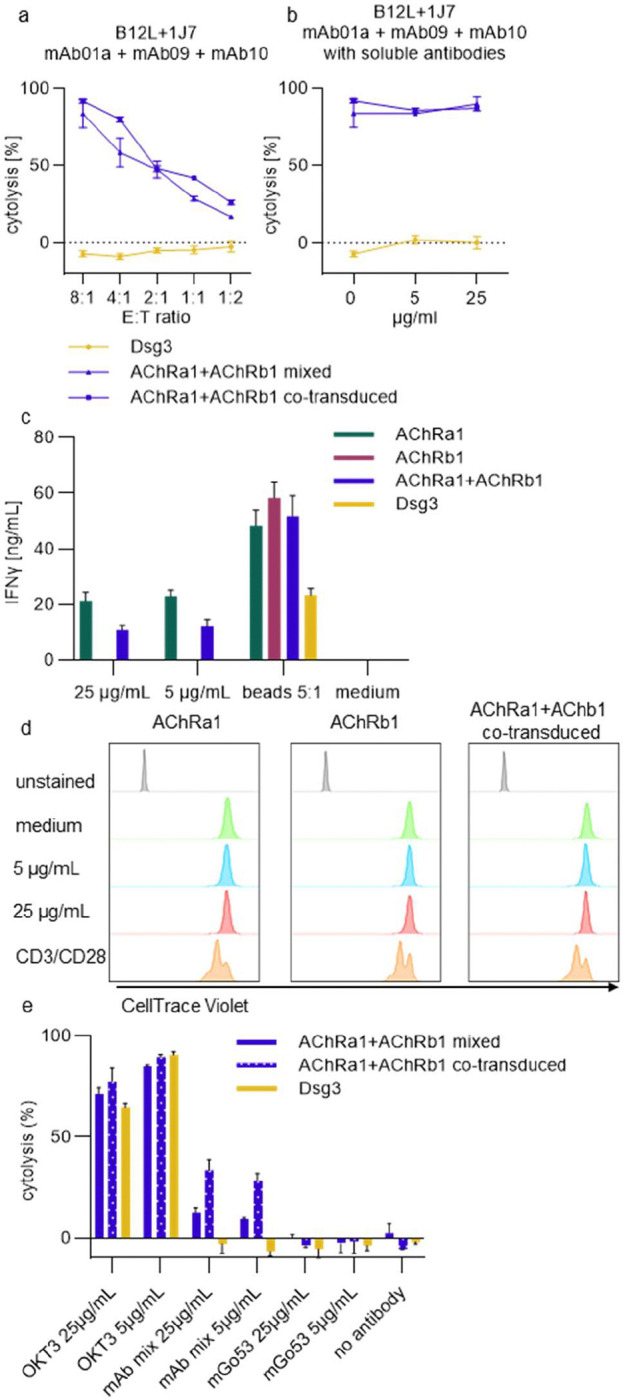
Soluble AChR autoantibodies do not impair killing efficiency but mediate low-level cytolysis of Fc receptor (FcR)-expressing cells. Selected functional assays were performed to evaluate the effects of soluble autoantibodies on AChR CAAR T cells. Monoclonal antibodies were supplemented with human polyclonal IgG (10 mg/mL) to mimic physiological serum antibody concentrations. (a–b) CAAR T cells co-transduced with AChRa1 and AChRb1 CAARs (co-transduced), or a mixture of CAAR T cells individually expressing AChRa1 or AChRb1 CAAR, were co-cultured for 20 hours with a mixture of AChR⍺1 and AChRβ1 target cells in the absence (a) or presence (b) of the corresponding soluble antibodies. E:T ratio in B was 8:1. Specific target cells cytolysis was measured via luciferase activity. Bars represent mean ± SD of triplicate cultures. (c) CAAR T cells were cultured without target cells for 20 hours in the presence of a mixture of AChR⍺1 and AChRβ1 autoantibodies at a total concentration of 25 μg/mL or 5 μg/mL. IFNγ levels were measured in the supernatants by ELISA. CD3/CD28 activation beads served as positive control. Bars represent mean ± SD of triplicate cultures. (d) Proliferation of AChRa1, AChRb1 or AChRa1+AChRb1 (co-transduced) CAAR T cells induced by a mixture of soluble anti-AChR autoantibodies after 72 hours was assessed by dilution of CellTrace Violet dye, analyzed via flow cytometry. CD3/CD28 activation beads served as positive control. (e) CAAR T cells were co-cultured with Fc receptor-positive (FcR^+^) THP-1 cells expressing firefly luciferase (ffluc) for 20 hours. The anti-CD3 antibody OKT3 served as a positive control. Specific cytolysis was measured via luciferase activity.

We first examined whether soluble AChR autoantibodies could inhibit AChR CAAR-mediated cytotoxicity. Co-transduced or mixed AChRa1 and AChRb1 CAAR T cells were co-cultured with a panel of AChRα1- and AChRβ1-reactive target cells in absence (Fig. 4a) or presence of the antibody mixture (Fig. 4b). Cytolytic activity was comparable between co-transduced and mixed CAAR T cells (Fig. 4a, 4b), and no reduction in target cell killing was observed in the presence of soluble autoantibodies (Fig. 4b), indicating that circulating AChR autoantibodies would do not block CAAR-BCR interactions.

#### Antibody-induced activation and proliferation.

Because AChR autoantibodies can dimerize AChR receptor subunits at the neuromuscular junction, soluble AChR autoantibodies may also be able to dimerize AChR-CAARs expressed on T cells. We thus tested whether soluble AChR autoantibodies might crosslink and activate AChR CAARs in the absence of target cells. AChR autoantibodies triggered modest IFNγ secretion by AChRa1 CAAR T cells alone, whereas AChRβ1-reactive autoantibodies alone did not induce cytokine release (Fig. 4c). Cytokine levels reached approximately 20–30% of those elicited by CD3/CD28 bead stimulation (Fig. 4c). No CAAR T cell proliferation was detected over 72 hours (Fig. 4d), further suggesting that autoantibody-induced signaling is transient and submaximal.

#### Fc receptor–dependent cytotoxicity.

To explore whether soluble AChR autoantibodies could bridge CAAR T cells to Fc receptor-bearing cells (such as natural killer cells or monocytes), we used THP-1 monocytic cells expressing type I and II Fc receptors in a luciferase-based killing assay. In the presence of AChR autoantibodies (5 or 25 μg/mL), CAAR T cells induced concentration-dependent cytolysis of THP-1 cells, which was more pronounced for co-transduced than for mixed CAAR T cells (Fig. 4e). However, maximal lysis remained approximately 70% lower than that induced by anti-CD3 antibody OKT3 (Fig. 4e). No cytotoxicity was observed with the irrelevant antibody mGo53 (Fig. 4e).

Together, these findings demonstrate that soluble AChR autoantibodies neither inhibit CAAR T cell function nor provoke sustained activation, although they can mediate minimal cytotoxicity toward Fc receptor-expressing cells in an artificial system. Relevance of this finding will be monitored in future clinical trials.

### AChR CAAR T cells control target cell outgrowth *in vivo* and restore pathological changes

To evaluate the *in vivo* activity of AChRa1 CAAR T cells, in a first step we used hybridoma cells expressing the pathogenic AChRα1-reactive autoantibody G10, transduced with firefly luciferase for bioluminescence tracking. After confirming efficient *in vitro* cytolysis (Supplementary Fig. 5a), 1×10^5^ G10 hybridoma cells were injected into NOG mice, followed by infusion of either AChRa1 CAAR T cells or untransduced T cells on day 2. Serial bioluminescence quantification revealed robust control of target cell outgrowth in three of five animals treated with AChRa1 CAAR T cells (Supplementary Fig. 5b, 5d), whereas all animals treated with untransduced T cells did not show target cell control (Supplementary Fig. 5b, 5c). One treated animal showed only partial target cell control with target cell regrowth starting from day 12 (Fig. S5b, S5d), albeit at markedly lower levels than controls. Serum antibody quantification paralleled these findings, with undetectable anti-AChR autoantibody levels in four treated mice compared to a mean of 54.8 μg/mL (range 9.7–101.6) in controls (p=0.0714, Supplementary Fig. 5e). Notably, the animal with partial target cell escape also lacked detectable serum autoantibodies, suggesting loss of antibody secretion and BCR downregulation.

AChRa1 CAAR T treatment also reversed some disease-associated changes at the neuromuscular junction (NMJ), as evidenced by a higher α-bungarotoxin signal intensity (mean MFI 4.04×10^6^ vs 2.51×10^6^, p=0.0476) reflecting increased AChR expression than in animals treated with untransduced T cells, which was associated with markedly reduced IgG deposition In three out of five animals (Supplementary Fig. 5f–h). Despite reduced AChR expression in animals treated with untransduced T cells no relevant changes of grip strength was observed compared to baseline values (Supplementary Fig. 5i). No signs of toxicity, weight loss, or behavioral changes were observed (Supplementary Fig. 5j). Together, these results demonstrate that AChRa1 CAAR T cells efficiently and safely deplete pathogenic B cells *in vivo*, leading to suppression of circulating and NMJ-bound autoantibodies.

To extend this approach to a polyclonal setting, in a second step we tested AChRa1 and AChRb1 CAAR T cells against a mixture of AChRα1- and AChRβ1-reactive Nalm6 cells (B12L and 6J2, respectively) previously characterized as pathogenic [5]. Both B12L and 6J2 Nalm6 cells expressed surface BCRs at physiological levels (Fig. 2) and secreted respective autoantibodies (Supplementary Fig. 6a), mimicking antibody-secreting cells. *In vitro*, co-transduced CAAR T cells secreted IFNγ and effectively lysed both target populations after co-culture (Supplementary Fig. 5j, 5k).

**Fig. 5.**
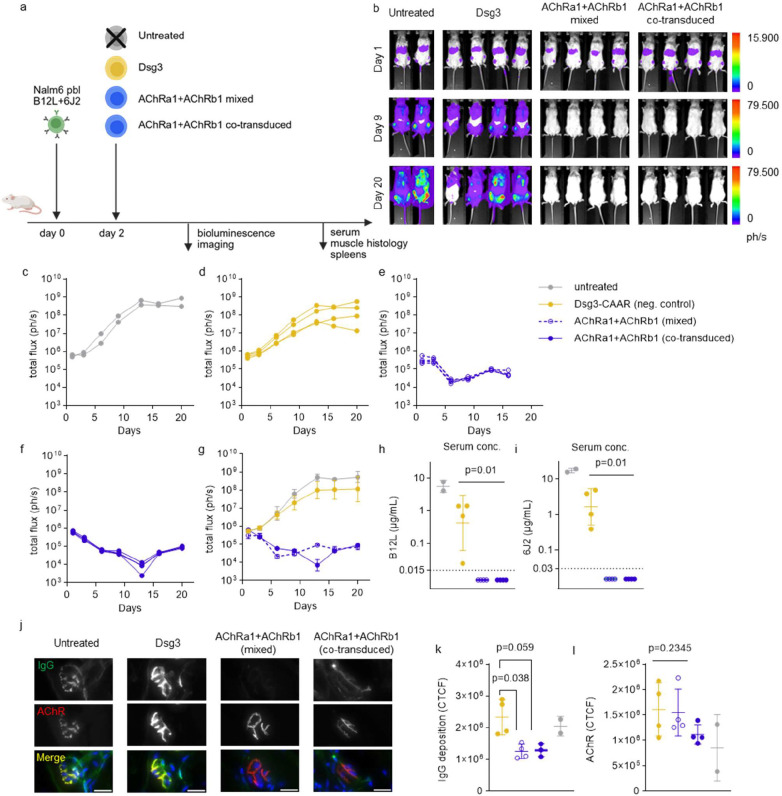
Depletion of AChR-autoantibody-expressing cells leads to undetectable antibody levels. AChR-autoantibody-secreting Nalm6 cells were engrafted into immunodeficient NOG mice, followed by treatment with PBS, Dsg3 CAAR T cells, or AChR CAAR T cells. Target cell burden was monitored by *in vivo* bioluminescence imaging throughout the study. (a) Schematic of the animal study design. (b) Representative bioluminescence images on days 1, 9, and 20 after target cell injection. (c–f) Total flux of individual mice treated with (c) PBS, (d) control Dsg3 CAAR T cells, (e) mixed AChRa1 and AChRb1 CAAR T cells, or (f) AChRa1 and AChRb1 co-transduced CAAR T cells. (g) Mean total flux ± SD for each treatment group. (h, i) Serum levels of B12L (h) and 6J2 (i) antibodies on day 21 measured by ELISA. Dashed lines indicate detection threshold. Scatter plots represent individual mice; bars indicate geometric mean ± geometric SD. (j) Confocal images of neuromuscular junctions showing IgG deposition and AChR expression by labeling with ⍺-bungarotoxin. Scale bar indicates 20 μm. (k, l) Quantitative analysis of (j). Scatter plots showing mean corrected total cell fluorescence (CTFC) per animal ± SD.

*In vivo*, NOG mice were injected with a 1:1 mixture of 1×10^6^ B12L and 6J2 cells, followed by infusion of either Dsg3 CAAR T cells, a 1:1 mixture of single-transduced AChRa1 and AChRb1 CAAR T cells, or co-transduced bi-specific CAAR T cells (Fig. 5a). Both CAAR T regimens markedly reduced bioluminescence signal starting from day 2 compared to controls (Fig. 5c–g), with similar efficacy between mixed and co-transduced T cells. Reductions in total flux was paralleled by a significant depletion of AChR-targeting B12L (p=0.0102; Fig. 5h) and 6J2 (p=0.0102; Fig. 5i) autoantibodies in serum and by reduction of IgG deposition at the NMJ (Fig. 5k). No significant differences in AChR expression were found compared to controls (Fig. 5l) and no relevant differences in grip strength were observed among groups up to day 20 (Supplementary Fig. 5l).

One co-transduced CAAR T cell-treated mouse was found dead on day 20, one day before the planned study endpoint. As all treatment groups displayed body weight loss from day 15 (Supplementary Fig. 6e–i), accompanied by T cell expansion in blood (Supplementary Fig. 6j) and spleen (Supplementary Fig. 6k), the death was most likely attributable to graft-versus-host disease, a known limitation of xenograft models.

These findings demonstrate that both mixed and co-transduced AChR CAAR T cells effectively eliminate AChRα1- and AChRβ1-reactive B cells *in vivo* while maintaining overall safety within the model constraints.

## DISCUSSION

We developed AChR CAAR T cells as a highly selective cellular therapy designed to eliminate autoreactive B cells specific for the AChRα1 and the AChRβ1 subunit of the AChR – the principal drivers of pathology in MG. AChR CAAR T cells demonstrated potent and specific cytolytic activity *in vitro* and *in vivo*, while maintaining stable surface expression and functional persistence. Importantly, we show that T cells can be co-transduced with distinct CAARs to simultaneously target multiple autoantibody specificities, establishing a versatile strategy for the treatment of autoimmune diseases with complex autoantigens.

In recent years, several novel therapies like complement inhibitors and neonatal Fc receptor (FcRn) inhibitors have improved disease control and reduced the frequency of myasthenic crises [9, 31]. However, these therapies do not target the underlying causal disease mechanism, typically do not induce remission and require chronic, potentially life-long, administration [9]. In contrast, AChR CAAR T cells were designed to achieve selective and deep depletion of autoreactive B cells producing pathogenic autoantibodies against AChRα1 and AChRβ1 subunits. This strategy leverages the potential of engineered T cells to eliminate tissue-resident B cells, which are often spared by conventional B cell-depleting agents such as rituximab [18, 32]. Furthermore, current therapies are largely non-selective and induce varying degrees of immunosuppression, increasing the risk of life-threatening infections and, in some cases, necessitating treatment discontinuation [33].

The selection of AChRα1 and AChRβ1 subunits for CAAR design was guided by their dominant roles in MG pathogenesis. AChRα1-specific autoantibodies are most frequently detected and are consistently the most pathogenic [2, 5, 6, 34, 35]. Recent studies have also highlighted the role of AChRβ1-specific autoantibodies, which contribute to pathogenesis through multiple mechanisms [5, 6, 24]. These findings underscore the rationale for targeting B cells producing AChRα1- and AChRβ1-specific autoantibodies to achieve maximal therapeutic benefit. Patient sera that bound to the AChRa1 CAAR predominantly came from individuals with symptomatic MG and high autoantibody titers suggesting that these patients may derive the greatest benefit from AChR CAAR T therapy. The combined AChRa1/AChRb1 CAAR approach captured the majority of patient sera tested, underscoring its ability to address polyclonal autoimmune responses.

Functionally, AChR CAAR T cells efficiently depleted AChRα1- and AChRβ1-reactive model B cells both *in vitro* and *in vivo*. We evaluated two approaches: a mixture of single-transduced CAAR T cells and co-transduced CAAR T cells expressing both receptors. While *in vitro* functionality was comparable, the co-transduced CAAR T cells demonstrated a trend toward superior target cell control *in vivo*. This finding suggests potential advantages of co-transduction for *in vivo* efficacy and has important implications for clinical manufacturing, as it enables a streamlined, single-process production approach, avoiding the need for parallel manufacturing and subsequent cell mixing.

Soluble AChR autoantibodies at concentrations typically found in serum of patients with MG did not inhibit CAAR T cell cytotoxicity but induced mild cytokine release and limited Fc receptor-dependent cytotoxicity. These effects were modest compared with full TCR activation and are consistent with preclinical data from Dsg3- and MuSK-CAAR T cell development, which have not resulted in severe cytokine release syndrome (CRS) in clinical application, based on early Phase I observations [36, 37]. Nevertheless, careful monitoring of FcR^+^ innate cell populations and cytokine profiles will be essential in future clinical translation.

*In vivo*, co-transduced CAAR T cells depleted a mixed population of AChRα1 and AChRβ1 target cells, with the same efficacy as a mixture of single-transduced CAAR T cells, leading to the depletion of autoantibodies both in serum and at the NMJ. These results support the ability of AChR CAAR T cells to disrupt a key pathomechanism driving MG and to function in the presence of circulating pathogenic autoantibodies. However, our mouse model did not permit evaluation of clinical disease reversal, as animals lacked a functional complement system and did not develop overt muscle weakness. Future studies using models with complement activity may enable direct assessment of clinical efficacy. Importantly, target cell outgrowth was stably controlled until the end of the experiment, consistent with the *in vitro* assessment showing no CAAR downregulation, differently from what has been previously reported for AChRa1 CAAR T cells [28]. The efficient engraftment, persistence, and stable CAAR expression demonstrated here will be crucial for the successful translation of this technology into clinical application.

Antigen-specific B cell depletion by CAAR T cells depends on B cell receptor (BCR) expression; however, long-lived plasma cells – which partially contribute to MG pathology, particularly in later stages of the disease – typically downregulate surface immunoglobulin expression. Although plasma cells may therefore evade direct targeting, the reported success of CD19 CAR T cell therapy in MG indicates that plasma cell depletion is not required to achieve deep and sustained remission [13–15].

Collectively, these findings position AChR CAAR T cells as a transformative precision immunotherapy for MG and support their advancement into first-in-human clinical evaluation.

### Limitations of the study

#### This study has the following limitations:

First, AChR CAAR T cells were not evaluated in an experimental autoimmune myasthenia gravis (EAMG) model, which would have enabled assessment of their therapeutic efficacy on the clinical phenotype and within the context of a polyclonal autoimmune response that more closely mirrors the human disease. Importantly, our *in vivo* model demonstrated not only efficient cytotoxicity of AChR CAAR T cells against target cells but also reversal of antibody-induced alterations at the neuromuscular junction, which can serve as a mechanistic proxy of MG pathology. Nonetheless, future studies employing immunocompetent EAMG models are warranted to determine whether AChR CAAR T cells can reverse disease manifestations *in vivo*.

Second, the efficacy of AChR CAAR T cells against primary B cells derived from MG patients was not directly tested *in vitro*. Such experiments would further validate their capacity to eliminate a polyclonal repertoire of autoreactive B cells. However, both serum and monoclonal autoantibody binding data support the concept that our CAAR constructs can recognize a broad spectrum of AChR-specific autoreactive B cell receptors.

Finally, the current AChR CAAR design does not target B cells reactive to alternative AChR subunits or to shared epitopes spanning multiple subunits [8]. Despite limited understanding of their pathogenic role [5, 6], future evidence of clinical relevance may warrant expansion of the AChR CAAR T platform to additional subunit specificities.

## METHODS

### RESOURCE AVAILABILITY

#### Lead Contact

Further information and requests for resources and reagents should be directed to and will be fulfilled by the Lead Contact, Niels von Wardenburg (niels.von-wardenburg@charite.de).

#### Materials Availability

All requests for materials including antibodies, viruses, plasmids, and proteins generated in this study should be directed to the Lead Contact author. Materials will be made available under a Material Transfer Agreement (MTA) for non-commercial usage.

#### Data and Code Availability

- CAAR construct sequences can be accessed at Genbank (accession number: PX776202, PX776203).
- This paper does not report original code.
- Any data or information required to reanalyze the data reported in this paper is available from the lead contact upon request.

### EXPERIMENTAL MODELS AND STUDY PARTICIPANT DETAILS

#### Myasthenia gravis patients and healthy donors

All primary samples (healthy donor and patient material) were obtained after informed consent and analyses were approved by the Institutional Review Board of Charité - Universitätsmedizin Berlin (study protocol number EA1/258/18), and the University of Florence/Careggi University Hospital (Florence, Italy) with protocol n. 22914_bio.

#### Cell lines

K562, Nalm6, Jurkat, and THP-1 cells were cultured at 37 °C in a humidified incubator with 5% CO_2_. K562, Nalm6, and Jurkat cells were maintained in RPMI 1640 medium supplemented with 10% fetal calf serum (FCS), 1% non-essential amino acids, 10 mM HEPES, and 1% penicillin/streptomycin. Cells were passaged twice weekly and maintained at densities of 4 × 10^5^ cells/mL (K562), 1 × 10^6^ cells/mL (Nalm6), and 1 × 10^5^ cells/mL (Jurkat). THP-1 cells were cultured in RPMI 1640 supplemented with 10% FCS, 1 mM sodium pyruvate, 50 μM 2-mercaptoethanol, 10 mM HEPES, and 1% penicillin/streptomycin, and passaged twice weekly to a density of 2 × 10^5^ cells/mL. HEK293T cells were maintained in Dulbecco’s Modified Eagle Medium (DMEM) supplemented with 10% FCS and 1% penicillin/streptomycin under the same incubation conditions.

#### T cells and lentiviral transduction

Peripheral blood mononuclear cells (PBMCs) were isolated by density gradient centrifugation and enriched for T cells through negative selection using a T Cell Isolation Kit (Miltenyi Biotec), following the manufacturer’s protocol. The isolated T cells were cultured in CTS^™^ OpTmizer^™^ T Cell Expansion Medium (Thermo Fisher Scientific), supplemented with GlutaMAX^™^, L-glutamine, and penicillin/streptomycin, and maintained at 37°C in a humidified incubator with 5% CO_2_. Interleukin-2 was added at a concentration of 50 IU/mL on days −1 (isolation), 0 (transduction), 1, 2, 5, 7, and 9. T cell activation was performed immediately after isolation using CD3/CD28 Dynabeads at a bead-to-cell ratio of 3:1. Cells were seeded at a density of 1 × 10^6^ cells/mL. On the following day, activated T cells were transduced using a spinoculation protocol. Protamine sulfate was freshly prepared at 1 mg/mL in PBS, sterile-filtered, and added to a final concentration of 10 μg/mL along with concentrated lentivirus. Cells were then centrifuged at 800 × g for 90 minutes at 32°C and incubated for two days at 37°C with 5% CO_2_. After transduction, T cells were cultured at a density of 0.5 × 10^6^ cells/mL, with cell density adjusted three times weekly. Cells were expanded over a period of 9 to 13 days before use in downstream experiments. Cell size was analyzed by an Invitrogen^™^ Countess^™^ automated cell counter.

#### Animal experiment approval and animal care

All animal procedures were conducted in accordance with the German Animal Welfare Act and approved by the appropriate regulatory authority (Federal Office for Health and Social Affairs, Berlin, Germany; approval number: vereinf. Genehm. E0023/23). Female NOD.Cg-*Prkdc*^*scid*^ *Il2rg*^*tm1Sug*^/JicTac mice (CIEA NOG mouse^®^), aged 6–8 weeks, were purchased from Taconic and housed under specific pathogen-free conditions. Animals were maintained on a 12-hour light/dark cycle at a constant temperature of 23°C, with food and water available ad libitum. Blinding was not applied in these experiments. The animal welfare was checked twice daily. General health conditions and body weights were recorded throughout the whole study.

## METHOD DETAILS

### AChR CAAR construct design

The CAAR construct sequences are available in GenBank under accession numbers PX776202 to PX776203. CAAR constructs include the ECDs of AChRα1 (amino acids 1–231) or AChRβ1 (amino acids 1–244), along with their respective endogenous signal peptides. These domains are connected via a flexible linker (amino acid sequence: ASGGGGSGGGGSSG) to a CD8α hinge region (accession no. NM_001768, amino acids 183–206), followed by a 4–1BB costimulatory domain (accession no. NM_001561, amino acids 214–255), and a CD3ζ signaling domain (accession no. NM_000734, amino acids 52–163). A short linker (LEGGG) and a T2A self-cleaving peptide sequence then precede a truncated epidermal growth factor receptor (EGFR) used for enrichment of CAAR-expressing cells. The transmembrane and intracellular domains are identical to those of the Dsg3-CAAR construct (accession no. KX348140).

### Lentivirus production

Lentivirus was produced as described previously [23].

### Production of recombinant AChR autoantibodies

The patient-derived monoclonal antibodies B12L, 1J7, 6J2, 5D2 and 3I3 were cloned, expressed, and purified as reported in [26] and [5]. The patient-derived monoclonal antibodies mAb01a, mAb01b, mAb02, mAb09 and mAb10 were cloned as reported in [6], while expression and IgG purification was performed as described in [38].

### ELISA-based detection of recombinant AChR monoclonal autoantibodies

Binding of recombinant monoclonal autoantibodies to the extracellular domains (ECDs) of AChRα1 and AChRβ1 was assessed using an ELISA-based approach as described previously [23, 39]. Rabbit Fc fusion proteins of the respective ECDs (AChRα1: amino acids 1–231; AChRβ1: amino acids 1–244) and of IgLON5 (NM_001101372; amino acids 1–314) were expressed in HEK293T cells. The Fc-tagged proteins were immobilized on 96-well plates via anti-rabbit IgG and the human monoclonal AChR antibodies were added. After extensive washing, bound antibodies were detected using HRP-conjugated anti-human IgG and 1-Step Ultra TMB substrate.

### Binding of recombinant autoantibodies and sera by flow cytometry

Jurkat T cells were transduced with lentiviral particles encoding AChRα1 or AChRβ1 CAAR constructs. For antibody binding assays, 1.5×10^5^ Jurkat cells (≈1:1 mixture of transduced and untransduced cells) were incubated with recombinant or monoclonal human antibodies (10 μg/mL) or sera (1:20) for 45 min on ice. Cells were then stained with goat anti-human IgG Fc (31125, Invitrogen; 1:200) for 20 min on ice, washed three times, and incubated with donkey anti-goat IgG (H+L) AlexaFluor-647 (1:800), cetuximab-PE anti-EGFR (1:10), and eBioscience^™^ Fixable Viability Dye eFluor^™^780 (1:500) for 20 min on ice. After washing, cells were resuspended in FACS buffer and analyzed using a FACSCanto II (BD Biosciences, NJ, USA) or MACSQuant Analyzer (Miltenyi Biotec, Germany). Fluorescence data were analyzed with FlowJo v10.9.0 and GraphPad Prism v9.5.1. IgG binding was quantified as the difference in geometric mean fluorescence intensity (ΔgMFI) between transduced (single, viable, cetuximab-PE^+^) and untransduced (single, viable, cetuximab-PE^−^) cells. The threshold for CAAR-binding sera was defined as the mean ΔgMFI of control samples+3 SDs. Binding of patient sera to full AChR (adult isoform) was assessed by live CBA flow cytometry as previously described [40, 41]. For validation of B12L and 6J2 antibody secretion by Nalm6 pbl cells, Jurkat cells expressing AChRα1 or AChRβ1 CAAR were incubated with undiluted culture supernatants for 20 min on ice and stained with anti-human IgG (A-21445, Invitrogen; 1:400) for 20 min on ice.

### Flow cytometry assessment of surface CAAR expression

Surface CAAR expression was assessed by flow cytometry on primary human T cells transduced with lentiviral particles encoding AChRa1 or AChRb1 CAAR on days 9, 12, and 15 of in vitro expansion. Briefly, 2×10^5^ AChRa1 CAAR-, AChRb1 CAAR-, or untransduced T cells were washed with PBS and incubated with mAb01a or mAb09 (10 μg/mL) for 45 min on ice. Cells were then washed and stained with secondary and tertiary antibodies and viability dye as described above, and analyzed on a FACSCanto II (BD Biosciences, NJ, USA). The percentage of surface CAAR-expressing cells among CD3^+^ T cells was quantified using FlowJo v10.9.0, and graphs were generated with GraphPad Prism v9.5.1.

### B cell receptor constructs and target cell lines

We engineered constructs for stable expression of human monoclonal antibodies in target cells, either as membrane-bound surface IgG or as secreted antibodies. Surface IgG expression was achieved by fusing the antibody to the immunoglobulin heavy chain (IgH) transmembrane domain and co-expressing the B-cell receptor (BCR) subunits CD79A and CD79B. For secreted antibody expression, constructs lacking the IgH transmembrane domain were used. To evaluate cytotoxicity against target cells, we utilized firefly luciferase (ffluc) tagged with green fluorescent protein (GFP) to enable both bioluminescent detection and cell sorting. Either the Nalm6 cells or K562 cells transduced with an ffluc-GFP lentivirus were used. The lentiviral construct pFUGW-Pol2-ffLuc2-eGFP (Addgene plasmid #71394), a gift from Glenn Merlino [17], was employed for this purpose. To generate target cell lines, Nalm6 ffluc-GFP and K562 cells stably expressing CD79A and CD79B were transduced with lentiviral vectors encoding the surface IgG construct and sorted based on surface human IgG expression using an anti-human IgG antibody coupled with Alexa Fluor^™^ 647 (1:400 dilution). For the generation of Nalm6 *pbl* cells – displaying the BCR on the cell surface while simultaneously secreting the corresponding antibody – Nalm6 cells initially expressing the surface-tethered antibody were further transduced with a version of the same antibody construct lacking the transmembrane domain. This enabled concurrent surface display and enhanced antibody secretion.

### B cell receptor density on target cells

To determine the BCR density on K562 and Nalm6 cells expressing surface IgG, number of BCR molecules was assessed using Quantum^™^ Alexa Fluor^®^ 647 MESF Kit (Bangs Labs) according to manufacturer’s instructions. Cell size was analyzed by a CASY Innovatis automated cell counter. For the comparison with human IgG-expressing B lymphocytes, PBMCs were isolated and then enriched for B lymphocytes by negative selection using a B cell isolation kit according to the manufacturer’s instructions (Miltenyi Biotec).

### Confocal Imaging of CAAR expressing Jurkat cells

AChRa1 and AChRb1 CAAR (co-transduced) expressing Jurkat cells were seeded into 15 μ-Slide 8 Well high glass-bottom dishes at a density of 5 × 10^5^ cells per well and incubated at 37 °C with 5% CO_2_ for 3 h. Cells were fixed with 4% paraformaldehyde (PFA) for 10 min at room temperature followed by washing with PBS. Cells were blocked for 1 h in blocking solution (20% BSA, 6% normal goat serum, 0.5% NaN_3_ in PBS) and stained with with mAb09 (10 μg/mL) and incubated overnight at 4 °C. On the following day, cells were washed three times with PBS and incubated with anti-human IgG labeled with Alexa Fluor 488 (used at 1:500) for 2 h at room temperature. Subsequently, cells were incubated with mAb35 (used at 10 μg/mL) overnight, followed by incubation with anti-rat IgG labeled with Alexa Fluor 594 (A11007, Invitrogen, used at 1:500) on the next day. Confocal images were acquired using the Nikon Spinning Disk Confocal CSU-X with an 60x Plan Apo λDIC N2 Oil NA 1.4 WD 130 μm objective. Images were taken at ~20°C with the Nikon elements 5.2 software.

### Enrichment of EGFRt^+^ CAAR T cells

EGFRt^+^ AChR-CAAR T cells were enriched as previously described [23]. In brief, CAAR T cells were purified on day 5–6 post-transduction using biotinylated cetuximab (used at 1:10) and anti-biotin MicroBeads, followed by magnetic selection (Miltenyi, OctoMACS or QuadroMACS depending on cell count). Remaining CD3/CD28 beads were removed prior to staining. Cells were resuspended at 0.5×10^6^/mL in T cell medium with 50 IU/mL IL-2.

### IFNγ and GrB release assays

For cytokine release assays 50,000 target target cells were co-cultivated with 50,000 CAAR T or control T cells in 200 μl RPMI medium supplemented with 10% FCS, 1% non-essential amino acids, 1 M HEPES, and 1% penicillin/streptomycin. After 24 hours, cell supernatants were collected following centrifugation. Human IFNγ levels were quantified using the Human IFN-γ DuoSet ELISA (R&D) at serial dilutions of 1:5, 1:25, 1:125, and 1:625. Granzyme B (GrB) release was measured using the Human Granzyme B ELISA BASIC kit (Mabtech) at the same dilution series. A modified protocol was used to assess IFNγ release induced by soluble antibodies. Instead of target cells a mixture of monoclonal antibodies (B12L, mAb01a, mAb02, mAb09, mAb10) was added to a final concentration of 25 μg/mL or 5 μg/mL. To model physiological serum conditions human intravenous immunoglobulins (IVIg; Kiovig, Takeda) was added to a final concentration of 10 mg/mL. CD3/CD28 beads at a 1:1 bead:target ratio served as positive control.

### CAAR activation assay

250,000 T cells were incubated with target cells at a 1:1 effector-to-target ratio in a total volume corresponding to 1×10^6^ cells/mL in a 24-well plate. Cells were cultured in RPMI medium supplemented with 10% FCS, 1% non-essential amino acids, 1 M HEPES, and 1% penicillin/streptomycin. After 16–20 hours, cells were stained on ice for 20 minutes in protein-free PBS with the following antibodies: cetuximab-AF647 (1:10), CD3-PerCP (1:20, clone UCHT1), CD69-BV510 (1:20, clone FN50), and Fixable Viability Dye eFluor 780 (1:100). After washing cells were resuspended in 200 μl of FACS buffer. Data were acquired on a FACSCanto II (BD Biosciences, NJ, USA) and analyzed using FlowJo version 10.6.

### Luciferase-based killing assays

For luciferase-based killing assays, cells were maintained in RPMI medium supplemented with 10% FCS, 1% non-essential amino acids, 1 M HEPES, and 1% penicillin/streptomycin. Serial dilutions of CAAR T cells in 50 μl volumes were plated into black Thermo Scientific^™^ Nunc^™^ F96 MicroWell^™^ plates containing 50 μl of target cells, resulting in a total volume of 100 μl. Co-cultures were set up with 200,000 T cells (an 8:1 effector-to-target ratio) or the respective serial dilutions, together with 25,000 target cells per well. After 16–20 hours, 100 μl of luciferase substrate was added to each row, and bioluminescence was measured using a SpectraMax iD3 reader. The percentage of viable cells was calculated by dividing the luminescence of each condition by that of the control wells containing target cells only.

A modified protocol was used to assess cytolysis of fluc-expressing FcR+ THP-1 cells induced by soluble antibodies. Target cells were resuspended in medium containing IVIg at a final concentration of 10 mg/mL to model physiological serum conditions, together with a mixture of monoclonal antibodies (B12L, mAb01a, mAb02, mAb09, mAb10) at a final concentration of 25 μg/mL or 5 μg/mL. CAAR T cells were then added at 8:1 effector-to-target ratio.

### Proliferation of AChR CAAR T cells

CAAR T cells and untransduced T cells were labeled with 5 μM CellTrace Violet according to the manufacturer’s instructions. Briefly, dye was diluted in PBS and cells were incubated for 20 min at 37 °C in the dark with gentle agitation. Labeled or unlabeled T cells were co-cultured for 6 days with K562 B12L, K562 mAb02, K562 6J2, K562 mAb09, a 1:1 mixture of K562 B12L and 6J2, or control K562 mGO53 cells at an E:T ratio of 5:1 (2.5×10^5^ T cells, 5×10^4^ target cells). CD3/CD28 beads (1:1 bead:target) served as a positive control. Medium was supplemented on day 3. On day 6, cells were stained with cetuximab-AF647 (1:10) and Fixable Viability Dye eFluor^™^780 (1:100), washed, resuspended in 200 μL FACS buffer, and acquired on a FACSCanto II (BD Biosciences, NJ, USA). Proliferation of CAAR-expressing T cells was determined by CellTrace Violet dilution and visualized as histograms using FlowJo v10.6.

To assess proliferation induced by soluble antibodies, target cells were replaced with a mixture of monoclonal antibodies (B12L, mAb01a, mAb02, mAb09, mAb10) at final concentrations of 25 or 5 μg/mL. To model physiological serum conditions, IVIg was added at 10 mg/mL. Cells were stained, acquired, and analyzed on day 3 as described above.

### Mouse model and bioluminescence quantification

To quantify target cells *in vivo*, serial bioluminescence imaging (BLI) of ffluc-expressing Nalm6 cells was performed after D-luciferin administration. Female NOD.Cg-*Prkdc*^*scid*^ *Il2rg*^*tm1Sug*^/JicTac mice (NOG, 6–8 weeks; Taconic) were maintained under specific pathogen-free conditions. To facilitate engraftment via Fcγ receptor blockade, mice received intraperitoneal IVIg (600 mg/kg) on days −2 and −1, with additional doses on imaging days [21]. On day 0, 0.8 × 10^6^ ffluc-expressing Nalm6 cells (1:1 mixture of Nalm6 pbl B12L and Nalm6 pbl 6J2; >98% ffluc-GFP^+^/surface IgG^+^) were injected intravenously, followed by 8 × 10^6^ CAAR T cells on day 2. BLI and body weight were recorded twice weekly under isoflurane anesthesia, and grip strength was measured five times per animal using a rodent grip strength meter (BIOSEB). In the experiment shown in Fig. 5, one mouse died on day 20, most likely due to graft-versus-host disease. BLI was performed 10 min after intraperitoneal injection of D-luciferin (150 mg/kg) using a NightOwl II LB983 system (Berthold Technologies) and IndiGO software (v2.0.5.0). Photon flux (ph/s) was quantified within a defined oval region of interest and corrected for background using a rectangular region without animals. Background correction resulted in negligible residual signal in IVIg-only control mice (no target cells; data not shown). Peripheral blood and serum were collected before sacrifice, and spleens were harvested *postmortem*. Serum AChR antibody concentrations were determined by ELISA as described below. Blood samples were collected via retrobulbar puncture into EDTA tubes (flow cytometry) or lithium-heparin tubes (serum). Serum was stored at −80 °C, while EDTA blood samples were analyzed immediately. Single-cell suspensions from spleens were generated by mechanical dissociation through a 70 μm strainer and resuspended in PBS containing 2 mM EDTA and 0.5% BSA. Spleen cells (100 μL) and EDTA blood (30 μL) were stained with antibodies against mouse CD45, human CD45, CD3, CD4, CD8, and 7-AAD for 10 min at 4 °C, followed by erythrocyte lysis (0.17 M NH_4_Cl, 10 mM KHCO_3_, 0.1 mM EDTA). Samples were analyzed on a MACSQuant16 (Miltenyi Biotec), and data were processed using FlowLogic software (Inivai Technologies). Total cell counts were calculated based on measured events, sample volumes, and total blood volume (estimated as body weight [g] × 0.074 mL/g). Spleen weights were used to determine cell counts per gram and per organ. All surviving animals were included in endpoint analyses.

Experiments with G10 hybridoma cells were performed similarly, with injection of 1 × 10^5^ hybridoma cells on day 0 followed by 1 × 10^7^ CAAR T cells or untransduced T cells

### ELISA of AChR antibodies in mouse samples

For quantification of G10, B12L or 6J2 antibody concentrations in mouse sera, Fc-tagged AChα1 or AChRβ1 extracellular domains (ECDs) were immobilized on 96-well plates via anti-rabbit IgG (10 μg/mL). Serum samples were diluted in StartingBlock (PBS) Blocking Buffer containing 0.05% Tween-20. Bound antibodies B12L/6J2 and G10 were detected using HRP-conjugated anti-human IgG or HRP-conjugated anti-mouse IgG, respectively, and developed with 1-Step Ultra TMB substrate. Antibody concentrations in sera were determined from calibration curves generated with purified B12L, 6J2 or G10.

### Histopathology of muscle tissue

The gastrocnemius muscle was fixated with 4 % PFA for at least one hour on ice after the preparation and stored for three days in a 30% sucrose-PBS solution at 4° C. After snap-freezing in N-Methylbutane, the muscles were cut into 20 μm thick longitudinal cryosections.

For immunofuorescent labeling the slides were thawed at room temperature and washed once with PBS. The sections were permeabilized for 10 min with 0,25 % Triton x100 in PBS and afterwards blocked for 30 min with 5 % NGS, 2 % BSA and 0,1 % Triton x100 in PBS at room temperature.

The secondary antibody goat anti-mouse IgG Alexa Fluor 488 (final conc. 3 μg/mL) was used to visualize antibody deposition at the postsynaptic site and combined with α-Bungarotoxin (final conc. 1 μg/mL) for AChR labeling. Sections were incubated at 4°C overnight. Finally, muscle sections were washed with PBS, labeled in PBS-containing DAPI (final conc. 2 μg/mL) and mounted with Immu-Mount^™^. Slides were imaged on a widefield microscope Leica SPE. Images were taken as Z-Stack scans using NIH ImageJ 1.52i software.

## QUANTIFICATION AND STATISTICAL ANALYSIS

All statistical analyses were performed using GraphPad Prism (version 10.5.0). Correlations between human serum AChR antibody titers measured by RIA and serum binding to AChRa1 or AChRb1 CAARs measured by flow cytometry (ΔgMFI; Fig. 1G and 1H, respectively) were assessed using two-tailed Spearman’s correlation and simple linear regression.

For in vivo bioluminescence experiments (Fig. 5G), data were analyzed using a mixed-effects model with post hoc Dunnett’s multiple-comparisons correction.

Nonparametric statistical tests were used for the comparison of serum antibody level geometric means among treatment groups. In Figures 5H and 5I, a Kruskal-Wallis test with Dunn’s correction was used. In Supplement Figure 5E a Mann-Whitney test was used. Values that were below the measurable range were set to one-half of the lower end of the measurable range in Figures 5H, 5I, and Supplementary Figure 5E. The Mann-Whitney test was used to perform the analyses shown in Figures 5K, 5L, and Supplementary Figures 5G and 5H.

Correlations between human serum binding strength (ΔgMFI) to the full adult isoform of the AChR and to AChRa1 or AChRb1 CAARs (Supplementary Fig. 1I and 1J, respectively) were evaluated using two-tailed Spearman’s correlation and simple linear regression. Comparisons shown in Supplementary Figures 5E, 5G, and 5H were analyzed using the Mann–Whitney U test. Grip strength and body weight were compared among treatment groups using the 2-way ANOVA (Supplementary Fig. 5I, 5J, 6D, 6E).

## Supplementary Material

Supplement 1

## INCLUSION AND DIVERSITY

We support inclusive, diverse, and equitable conduct of research.
