# Supplementary material for: Engineered Bi-Specific AChR CAAR T Cells for Selective Elimination of Myasthenia Gravis B Cells
Source: Res Sq. 2026 Mar 20:rs.3.rs-9094407. Preprint. [Version 1] doi: 10.21203/rs.3.rs-9094407/v1 (PMC13015596; doi:10.21203/rs.3.rs-9094407/v1)
Supplement: Supplement 1 [file NIHPPrs9094407v1-supplement-1.pdf]

SUPPLEMENTAL FIGURES AND LEGENDS

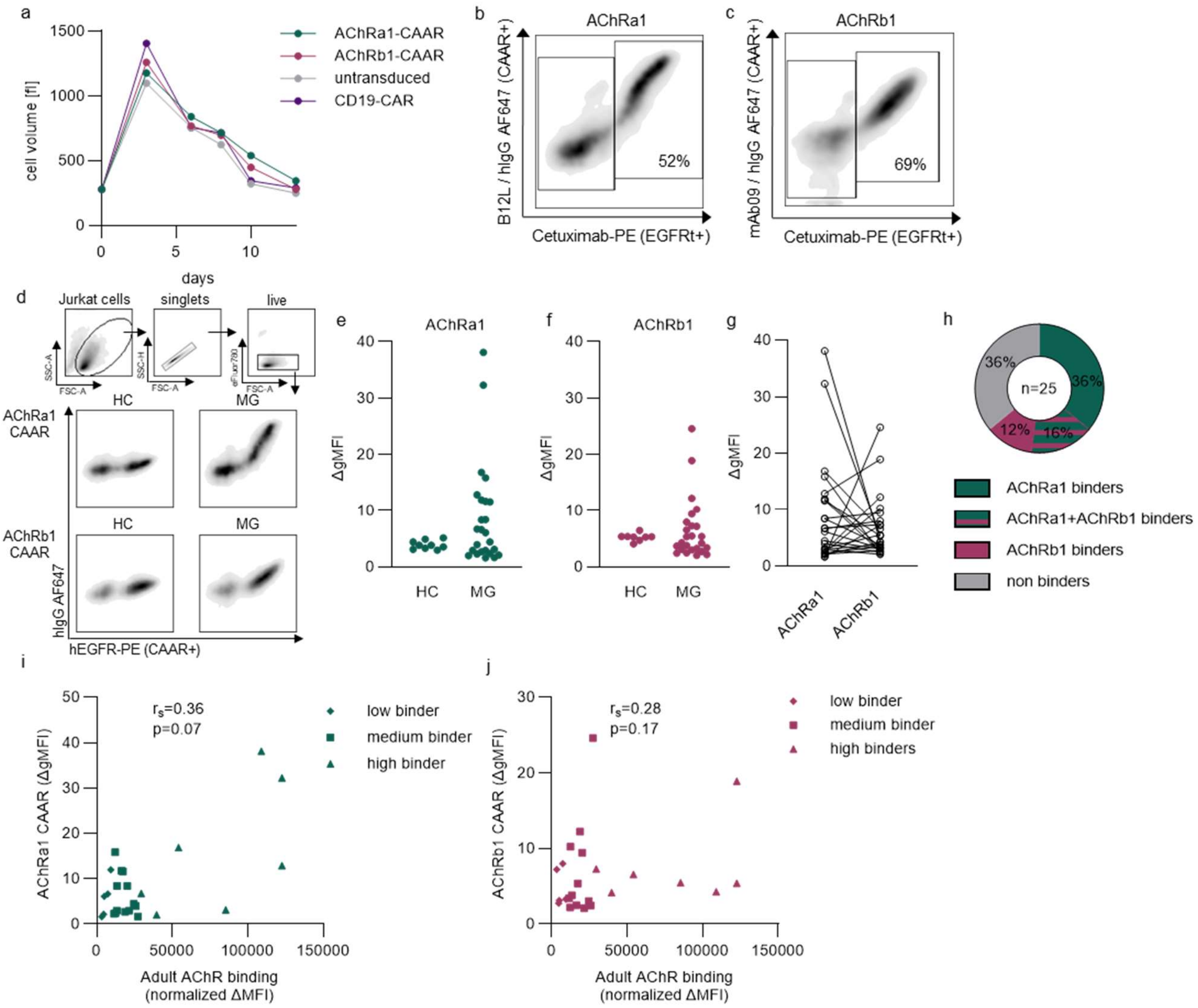

Supplementary Fig. 1. Validation of CAAR expression by Jurkat cells and MG serum binding, related to Fig. 1

(a) T cell volume was determined by an automated cell counter.

(b and c) Density plots showing tEGFR and CAAR expression in Jurkat cells. AChRa1 CAAR (B) and AChRb1 CAAR (C) expression was tested via flow cytometry by B12L (anti-AChRα1) and mab09 (anti-AChRβ1) binding, respectively.

(d-f) IgG binding of MG and HC serum on AChR CAAR T cells. (d) indicates the gating strategy. Binding to AChRa1 (e) or AChRb1 (f) CAAR expressed on Jurkat cells was assessed by flow cytometry. Scatter plots indicate geometric mean fluorescence intensity (gMFI) of EGFR+ cells minus gMFI of EGFR- cells (ΔgMFI). Error bar represents mean.

(g) Scatter plots indicate ΔgMFI of MG serum as in (e) and (f). Connecting lines illustrate data points from individual patients.

(h) Percentage of binders to AChRa1 CAAR (green), AChRb1 CAAR (red), AChRa1 CAAR and AChRb1 CAAR (red and green) or non-binders (gray) are shown in a donut chart. Cut-off for binding was defined based on the mean ΔgMFI of control samples + 3 SDs.

(i and j) Correlation of serum binding to AChRa1 CAAR (i) and AChRb1 CAAR (j) with binding to full AChR receptor as measured by RIA. Statistical analysis was performed by two-tailed Spearman’s correlation.

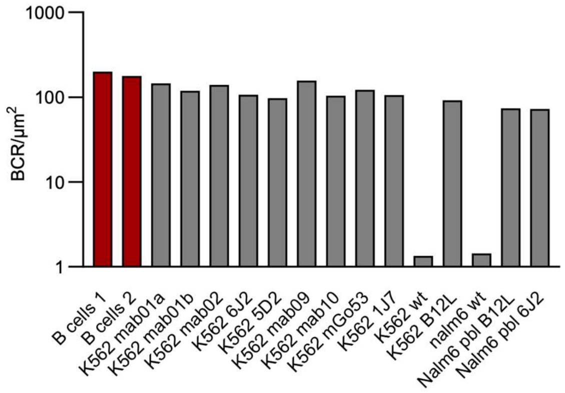

Supplementary Fig. 2. Validation of BCR expression by target cells and changes in CAAR T cell size during expansion, related to Fig. 2

Bar diagramm showing B cell receptor density based on monoclonal anti-human-IgG antibody binding as determined by flow cytometry. Quantification was performed using APC quanitification beads. Cell surface was determined by an automated cell counter.

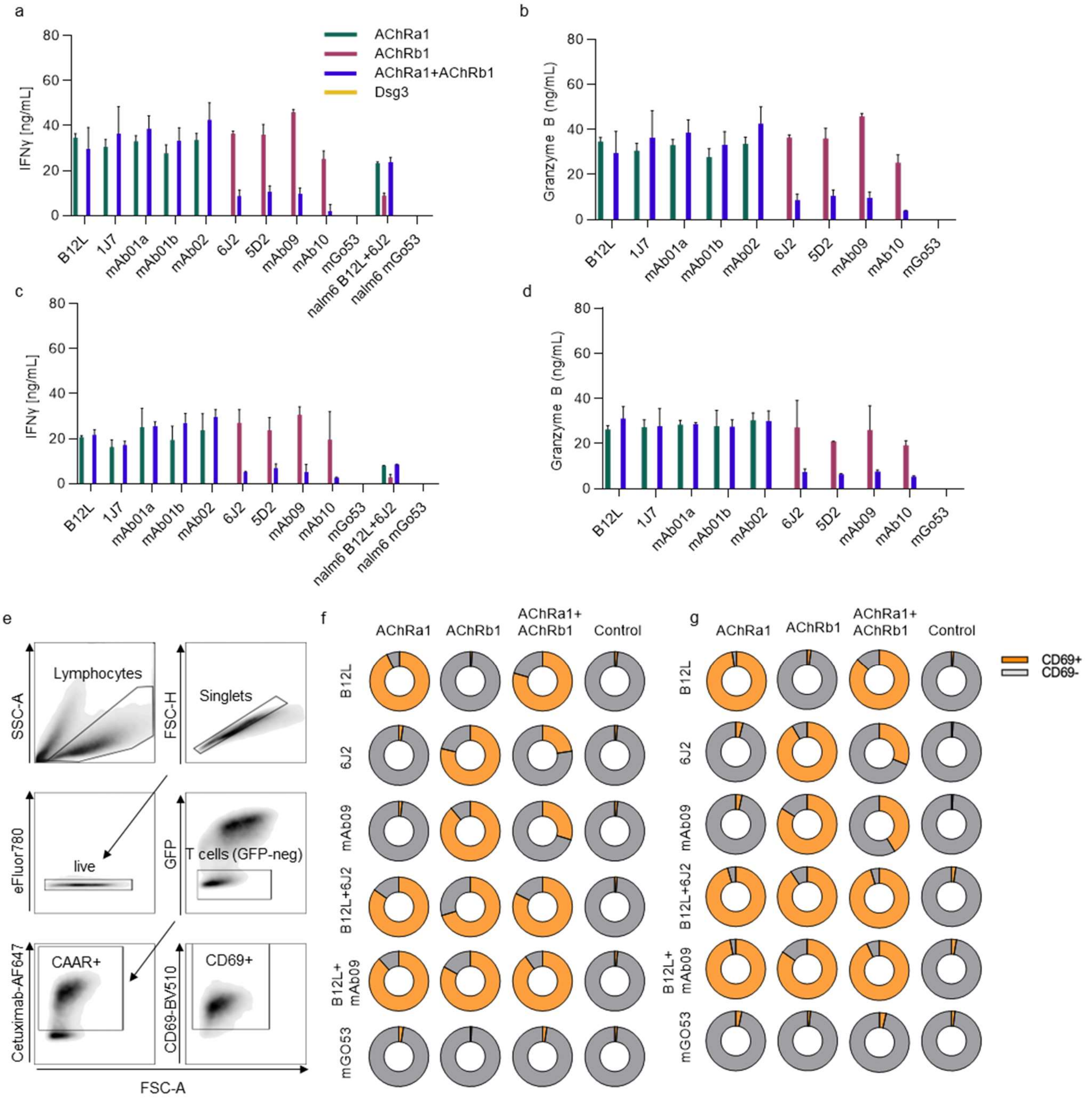

Supplementary Fig. 3. Target cell-induced secretion of effector molecules and expression of activation marker, related to Fig. 2

Donor-derived CAAR T cells expressing AChRa1, AChRb1, or co-transduced AChRa1+AChRb1 were co-cultured with K562 cells engineered to express AChRa1- or AChRb1-specific autoantibodies as surface B cell receptors (BCRs), to assess T cell activation and proliferation.

(a-d) After 24 hours of co-culture of CAAR T cells from two different donors, supernatants were collected and analyzed by ELISA for IFNγ (a, c) and GrB (b, d) levels. Bars represent mean ± SD of triplicate cultures.

(e) Gating strategy used to determine percentage of CD69+ CAAR T cells.

(f, g) Pie charts showing the percentage of CAAR T cells of two different donors expressing the early activation marker CD69 after 20 hours of co-culture, as determined by flow cytometry.

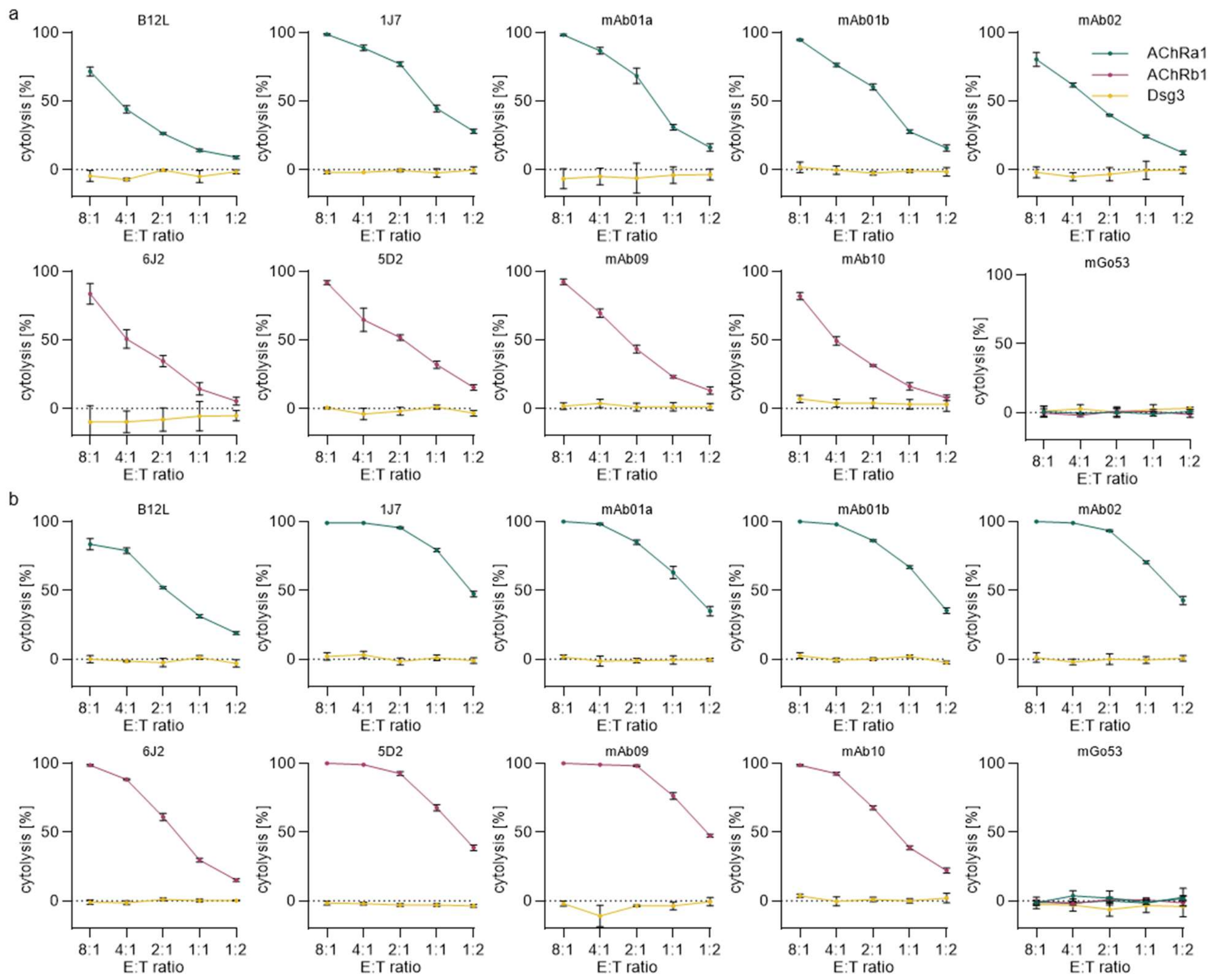

480

481 **Supplementary Fig. S4. AChR CAAR T cells eliminate target cells in-vitro, related to Fig. 3**

482 (a, b) AChR CAAR T cells from two differents donors were incubated with ffluc-expressing target cell lines for 16-20 hours,
483 followed by measurement of specific cytolysis via luciferase activity. Mean values ± SD of triplicate cultures are shown.
484 Dsg3 CAAR T cells were included as negative controls.

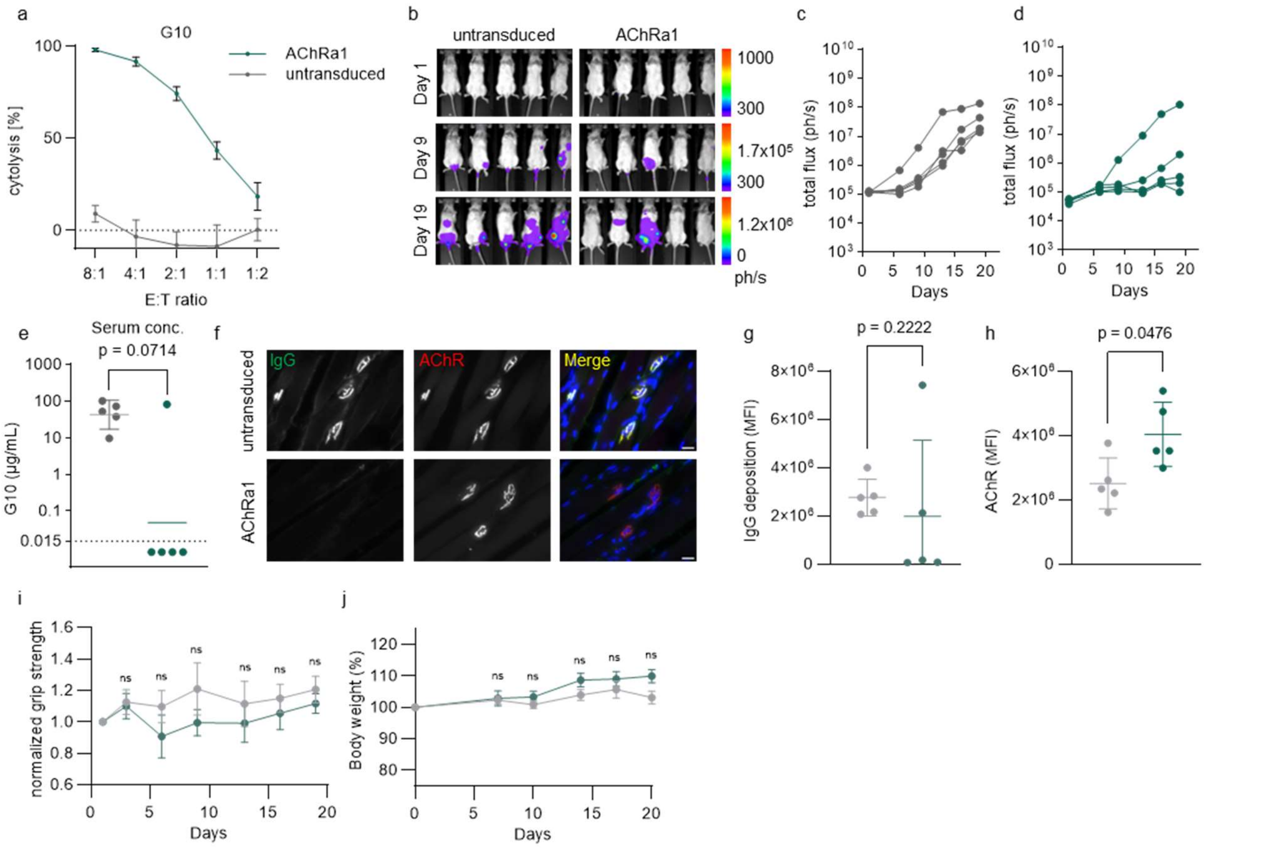

Supplementary Fig. S5. AChRa1 CAAR T cells eliminate G10 hybridoma cells and prevent AChR downregulation, related to Fig. 5

(a) AChRa1 CAAR T cells were incubated with ffluc-expressing and AChRα1-antibody secreting G10 hybridoma cells for 16-20 hours, followed by measurement of specific cytolysis via luciferase activity. Mean values ± SD of triplicate cultures are shown. Untransduced T cells were included as negative controls.

(b-h) G10 hybridoma cells were engrafted into immunodeficient NOG mice, followed by treatment with AChRa1 CAAR T cells. Target cell burden was monitored by *in vivo* bioluminescence imaging throughout the study and immunohistological analysis of gastrocnemius muscle was performed *postmortem*.

(b) Representative bioluminescence images on days 1, 9, and 20 after target cell injection.

(c, d) Serial quantification of G10 hybridoma cell burden by bioluminescence imaging. Total flux of individual mice treated with untransduced T cells (c) or AChRa1 CAAR T cells (d).

(e) Serum levels (day 24) of G10 antibody as quantified by ELISA. Scatter plots show values of individual animals and geometric mean ± geometric SD. No error bars are shown for groups with values below the measurable range.

(f) Representative immunohistochemical images of gastrocnemius muscle stained. Images show IgG deposition (left column), AChR (middle column) and the merged composite of mice treated with untransduced T cells (top) or AChRa1 CAAR T cells (bottom). AChR was labeled by α-bungarotoxin.

(g, h) Scatter plots showing mean MFI per animal of IgG deposition (G) or AChR (H) + SD. Lines indicate median.

(i) Mean grip strength ± SEM of individual animals are shown. For each animal, values were normalized to grip strength determined on the day of target cell injection.

(j) Mean body weights ± SEM of individual animals are shown. For each animal, values were normalized to bodyweight determined the day before target cell injection.

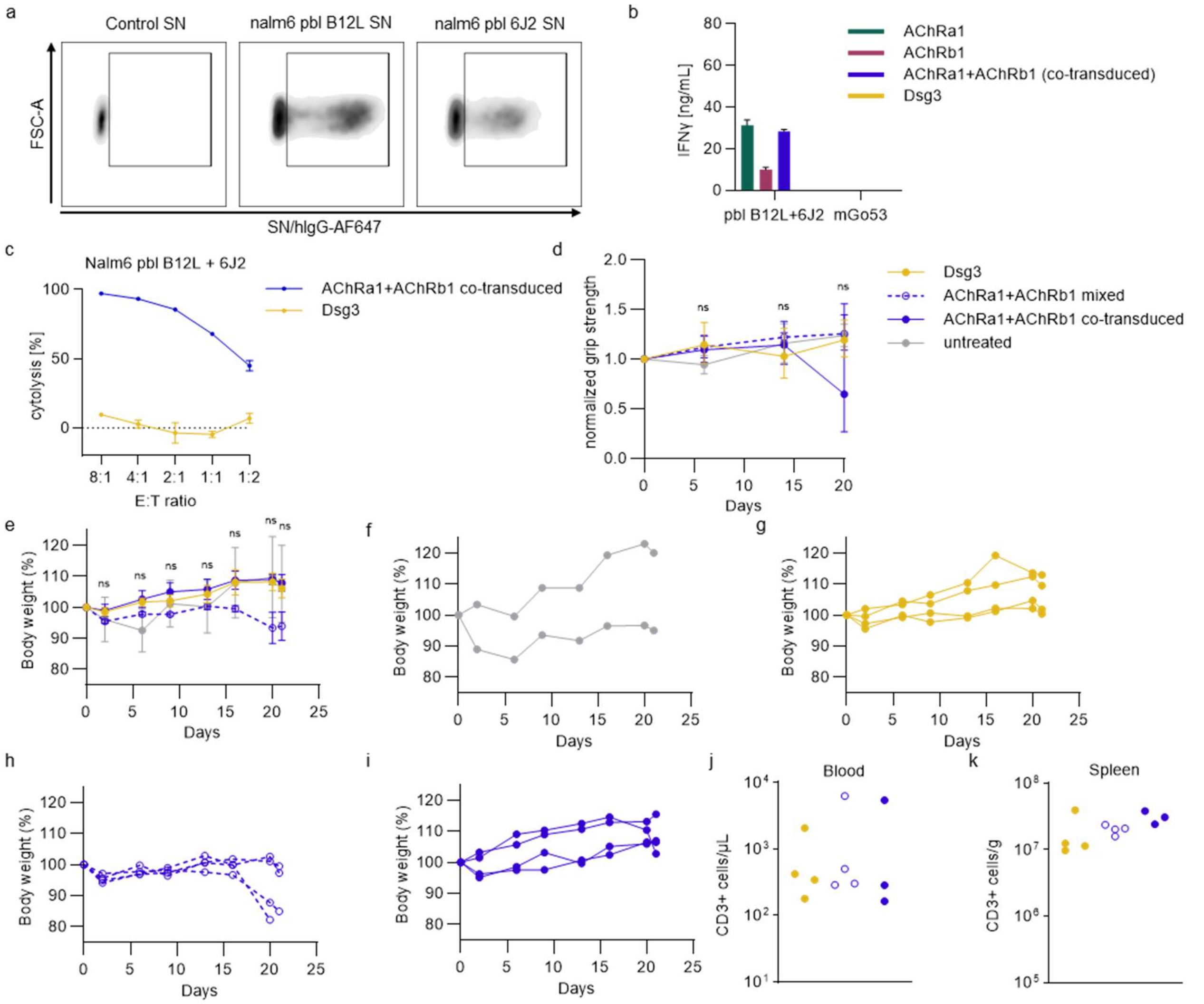

Supplementary Fig. S6. Antibody-secreting target cells and clinical evaluation of animals treated with AChR CAAR T cells, related to Fig. 5

(a-c) Nalm6 sIg B12L and sIg 6J2 were transduced to also secrete the respective antibody. These cells were named Nalm6 pbl B12L and Nalm6 pbl 6J2.

(a) Antibody secretion was validated by flow cytometry assessment of cell culture supernatant of nalm6 pbl B12L and nalm6 pbl 6J2 on Jurkat cells co-expressing AChRa1 CAAR and AChRb1 CAAR.

(b) AChR CAAR T were co-cultured with a 1:1 mixture of Nalm6 pbl B12L and Nalm6 pbl 6J2 cells. After 24 hours of co-culture, supernatants were collected and analyzed by ELISA for IFNγ. Bars represent mean ± SD of triplicate cultures.

(c) AChR CAAR T cells were incubated with with a 1:1 mixture of Nalm6 pbl B12L and Nalm6 pbl 6J2 cells for 16-20 hours, followed by measurement of specific cytolysis via luciferase activity. Mean values ± SD of triplicate cultures are shown.

(d) Grip strength was measured four times during the study period. Mean body weights ± SD of individual animals are reported. For each animal, values were normalized to grip strength determined the day before target cell injection.

(e) Bodyweight was measured on days of bioluminescence measurements. Mean body weights ± SD of individual animals are reported. For each animal, values were normalized to bodyweight determined the day before target cell injection.

(f-j) Body weight of individual (f) untreated animals, animals treated with (g) Dsg3 CAAR T cells, (h) mixed AChRa1 and AChRb1 CAAR T cells, or (i) AChRa1 and AChRb1 co-transduced CAAR T cells or (j) animals without target cell injection and only IVIg treatment.

(k, l) Scatter plots showing CD3+ T cell counts in peripheral blood and in spleen as assessed by flow cytometry.

SUPPLEMENTAL TABLE TITLES AND LEGENDS

Supplementary Table 1. Characteristics of recombinant AChR autoantibodies. Related to all Figures.

mAb ID	Patient characteristics	Source	Specificity and isotype	Antigenic modulation	Complement activation	Direct blocking	*In vivo* pathogenicity
B12L	34y, M; gMG, MGFA= IVb	MBC[2]	α1, IgG1	yes	n.a.[1]	no	Yes (rat passive transfer MG model)
1J7	39y, F; gMG	CD19+ B cell[3]	α1, IgG1	yes[4]	yes	no	Yes, when combined with 6J2 (rat passive transfer MG model)
6J2	63y, M; oMG	CD19+ B cell[3]	β1, IgG4[5]	yes[4]	yes, in combination with B12L	no	Yes, when combined with 1J7 (rat passive transfer MG model)
5D2		CD19+ B cell[3]	β1, IgG4	n.a.	yes, in combination with B12L	no	n.a.
3I3	35y, M; gMG, thymoma	CD19+ B cell[3]	β1, IgG3	n.a.	yes	no	n.a.
mAb01a	21-25y, F; EOMG	B cells[6]	α1	yes	yes	yes[7]	n.a.
mAb01b		B cells[6]	α1	yes	yes	yes	n.a.
mAb02	36-40y, F; EOMG	B cells[6]	α1	yes	yes	yes	n.a.
mAb09	86-90y, LOMG; MGFA=IVb	B cells[6]	β1	yes	yes	yes	n.a.
mAb10	46-50y, F; EOMG, MGFA=IIIb	B cells[6]	β1	yes	yes	no	n.a.

gMG= generalized myasthenia gravis; MBC=memory B cell; n.a.= not assessed; oMG= ocular myasthenia gravis;

1=not assessed in vitro; evidence of C3 deposition colocalizing with a-BTX labelled nAChR at the NMJs at the histopathological analysis of the rat passive transfer model.

2=sorted after alfa1-ECD-PE bait from the CD19++,IgG++,antigen++ gate.

3= antigen-specific B cells isolated with the membrane-antigen-capture activated cell sorting (MACACS) technique, after magnetic enrichment of B cells from PBMC.

4= nAChR reduction at the NMJ histopathological analysis (aBTX staining) in the passive transfer rat model.

5= cloned into an IgG1 backbone and expressed as IgG1 for in vitro and in vivo functional studies.

6= magnetic beads-enriched B cells cultured and differentiated in vitro into antibody-secreting cells.

7=as suggested by competition with aBTX, direct receptor blocking not demonstrated with electrophysiological studies.

Supplementary Table 2. Clinical and demographic characteristics of MG patients included in the study. Related to Figure 1 and Supplementary Figure 1

**Cohort 1 – Patients with high AChR autoantibody titers**	
N. of patients	17
Median age at MG onset (IQR), years	52 (27.5-62.5)
N. of female patients (%)	9 (53%)
N. of patients with thymoma (%)	8 (47%)
**Maximum MGFA grade, n. (%)[a]**	
I	1 (6%)
II	5 (31%)
III	2 (13%)
IV	4 (25%)
V	5 (31%)
**Immunotherapy at sampling, n. (%)[a]**	
none	6 (38%)
prednisone	3 (19%)
prednisone + azathioprine	4 (25%)
azathioprine	2 (13%)
prednisone, azathioprine, eculizumab	1 (6%)
**MGFA post-intervention status (PIS) at sampling, n. (%)**	
Minimal manifestations-or-better	6 (35%)
Symptomatic (MGFA-PIS= improved, unchanged, worse)	11 (65%)
**Cohort 2 – Patients with mixed AChR autoantibody titers**	
N. of patients	25
Median age at MG onset (IQR), years	63 (49.5-70.5)
N. of female patients (%)	8 (32%)
N. of patients with thymoma (%)	10 (40%)
**Maximum MGFA grade, n. (%)**	
I	8 (32%)
II	10 (40%)
III	7 (28%)
IV	0
V	0
**Immunotherapy at sampling, n. (%)**	
none	8 (32%)
prednisone	11 (44%)
prednisone + azathioprine	3 (12%)
prednisone + mycophenolate mofetil	2 (8%)
**MGFA post-intervention status (PIS) at sampling, n. (%)**	
Minimal manifestations-or-better	14 (56%)
Symptomatic (MGFA-PIS= I, U, W)	11 (44%)

a =not available in one patient.
